# Successful In Vitro Modification of the *Dmd* Gene Using Prime Editing

**DOI:** 10.3390/cells15090740

**Published:** 2026-04-22

**Authors:** Ayesha Siddika, Fatima El Husseiny, Joël Rousseau, Jacques P. Tremblay

**Affiliations:** 1 Département de Médecine Moléculaire, Université Laval, Quebec City, QC G1V 0A6, Canada; ayesha.siddika@crchudequebec.ulaval.ca (A.S.); fatima.el-husseiny.1@ulaval.ca (F.E.H.); 2 Centre de Recherche du Centre Hospitalier Universitaire de Québec, Quebec City, QC G1E 6W2, Canada; joel.rousseau@crchudequebec.ulaval.ca

**Keywords:** prime editing, Duchenne muscular dystrophy, *Dmd* gene, myogenic cells, CRISPR/Cas9, RTT–PBS optimization, point mutation, genome editing

## Abstract

**Highlights:**

**What are the main findings?**

- A 5′-TTCT-3′ motif in epegRNAs limits prime editing efficiency at the mdx-4cv and mdx-5cv *Dmd* loci.
- Silent substitution of this motif enhances editing efficiency up to 20–21% in C2C12 myoblasts.

**What are the implications of the main findings?**

- epegRNA sequence features play a critical role in determining prime editing efficiency.
- Optimized epegRNAs provide a valuable framework for therapeutic DMD correction in both in vitro and in vivo studies.

**Abstract:**

Duchenne muscular dystrophy (DMD) is a fatal X-linked neuromuscular disorder caused by mutations in the dystrophin gene. Prime editing is a versatile genome editing technology capable of introducing precise nucleotide changes without generating double-strand DNA breaks, making it a promising approach for correcting pathogenic point mutations. In this study, we applied prime editing to modify mdx-4cv and mdx-5cv mutation-equivalent sites in mouse C2C12 myoblasts in vitro. Initial editing efficiencies were unexpectedly low and were associated with the presence of a 5′-TTCT-3′ motif within engineered prime editing guide RNAs (epegRNAs). epegRNA designs containing this motif exhibited reduced prime editing efficiency, whereas silent substitution eliminating the motif significantly improved editing outcomes, indicating that specific sequence features within epegRNAs can influence editing performance. Rational redesign of epegRNAs to remove this motif substantially enhanced editing efficiency, achieving up to 20% modification at the 4cv target site using an NGG PAM and 21% editing at the 5cv locus using an NGAG PAM. These findings highlight an important sequence-dependent constraint in epegRNA design and provide practical guidance for optimizing prime editing strategies targeting *Dmd* mutations in vitro.

## 1. Introduction

Gene therapy has emerged as a transformative approach for the treatment of inherited genetic disorders by directly addressing pathogenic DNA variants rather than alleviating downstream symptoms [1,2]. Precise genome editing strategies offer the possibility of durable and potentially curative interventions through permanent modification of disease-causing mutations [3,4,5]. However, the clinical translation of genome editing technologies depends critically on editing precision, efficiency, and minimization of unintended genomic damage.

Early gene editing platforms, including zinc-finger nucleases (ZFNs) and transcription activator-like effector nucleases (TALENs), enabled locus-specific DNA cleavage but were limited by complex design requirements and the induction of double-strand DNA breaks (DSBs) [6,7]. The introduction of the CRISPR/Cas9 system in 2012 represented a major breakthrough, providing a simple and programmable platform for genome editing [8]. Nevertheless, conventional CRISPR/Cas9 editing relies on DSBs, which can trigger error-prone repair pathways and generate insertions, deletions, or chromosomal rearrangements.

To overcome these limitations, next-generation genome editing technologies were developed, including base editors capable of targeted nucleotide transitions without DSBs [9,10,11]. While base editing has enabled correction of many pathogenic variants, its applicability is restricted to specific base substitutions and constrained by editing windows and bystander edits.

**Figure 1:**
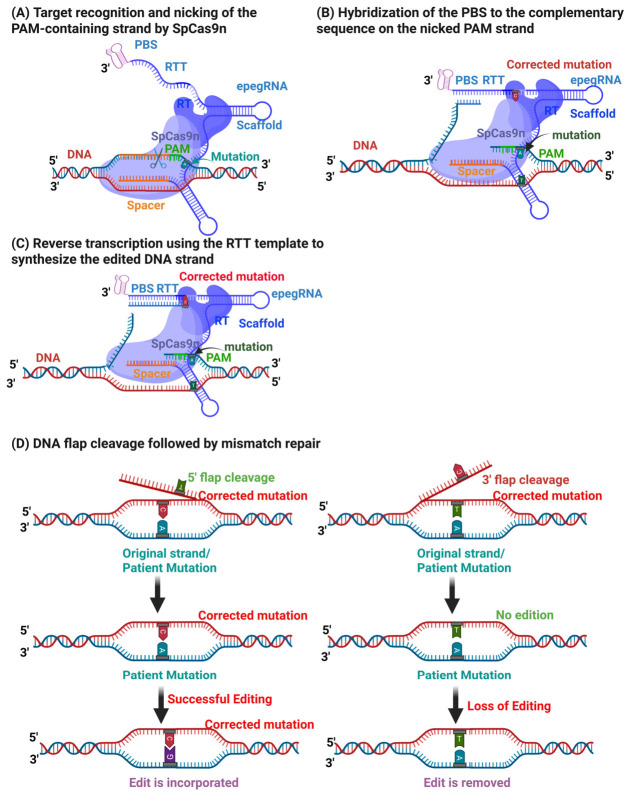
**Prime editing mechanism.** (**A**) The spacer sequence of the epegRNA binds to its complementary genomic sequence, directing the Prime Editor to the target site. Cas9 nickase (Cas9n) recognizes the PAM sequence and introduces a single strand cut 3 nucleotides upstream. (**B**) The primer binding site (PBS) anneals to the complementary sequence on the nicked DNA strand. (**C**) The reverse transcription template (RTT) serves as a template to synthesize the edited DNA strand. (**D**) The resulting DNA flap is resolved, and any mismatches are repaired, completing the edit. Image created with Created in https://BioRender.com.

Prime editing, first reported in 2019, represents a further advance by enabling targeted insertions, deletions, and all twelve possible base-to-base conversions without introducing DSBs or requiring donor DNA templates [12,13] (Figure 1).

Following nicking of the target strand, the primer binding site (PBS) anneals to the exposed DNA strand, allowing reverse transcription of the reverse transcription template (RTT) and installation of the edit through endogenous DNA repair pathways (Figure 1). Subsequent refinements, including the PE3 strategy and engineered pegRNAs (epegRNAs), have further improved editing efficiency by stabilizing guide RNAs and biasing DNA repair outcomes [12,14].

**Figure 2:**
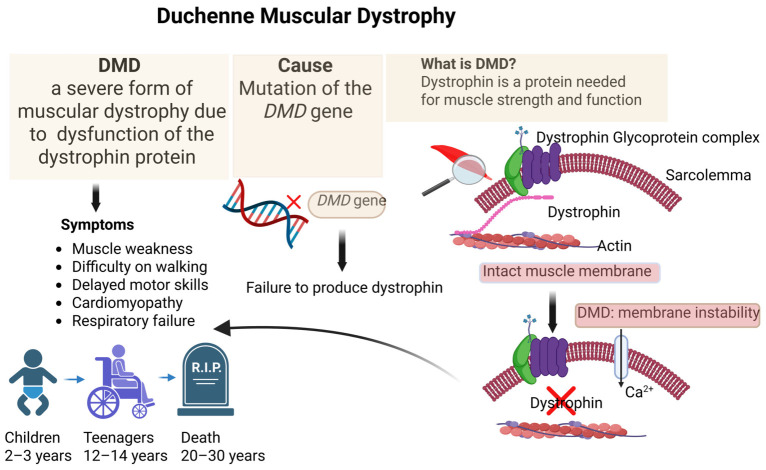
**Duchenne Muscular Dystrophy: cause and symptoms**. Image created with https://BioRender.com.

Duchenne muscular dystrophy (DMD) is a severe X-linked recessive neuromuscular disorder caused by mutations in the dystrophin gene (*DMD*) [15]. Loss of dystrophin compromises sarcolemmal stability, leading to progressive muscle degeneration, inflammation, fibrosis, and eventual cardiopulmonary failure [15,16,17] (Figure 2). Despite advances in supportive care and the approval of exon-skipping therapies and micro-dystrophin gene replacement strategies, DMD remains incurable, and existing treatments are mutation-specific or provide only partial functional benefit [17,18,19,20,21,22,23,24,25].

Importantly, approximately 30% of DMD cases arise from single-nucleotide variants, making them attractive candidates for precise genome correction strategies [17]. Prime editing is particularly well suited for correction of such mutations, as it enables exact restoration of the wild-type sequence without introducing frameshifts or large genomic alterations.

In this study, we investigated prime editing-mediated modification of two well-characterized DMD point mutations, mdx-4cv and mdx-5cv [26,27,28], using mouse myoblasts as an in vitro model. Since these models harbor single-nucleotide point mutations, we employed prime editing as a precise genome-editing strategy [29,30]. During the establishment of editing strategies for these loci, we identified a previously underappreciated constraint in engineered prime editing guide RNA (epegRNA) design involving a short transcription termination motif that markedly limited editing efficiency. Through rational redesign of epegRNAs, we achieved efficient modification of both mutations in vitro, highlighting key design considerations relevant to the future development of prime editing-based therapeutic strategies.

## 2. Materials and Methods

### 2.1. Cell Lines

The mouse myoblast cell line C2C12 was obtained from the American Type Culture Collection (ATCC, Manassas, VA, USA). This cell line was originally derived from the skeletal muscle of a C3H mouse following a crush injury and is widely used as an in vitro model to study myogenesis, muscle regeneration, and neuromuscular disease mechanisms. C2C12 cells retain a stable diploid genome and are amenable to genetic manipulation, making them suitable for evaluating genome-editing strategies targeting muscle-associated genes such as *Dmd*. All experiments were performed using low-passage cells to minimize genetic drift and phenotypic variability.

### 2.2. Plasmids

Prime editing plasmids pCMV-PE2 (Addgene #132775), pCMV-PE6a (Addgene #207851), pCMV-PE2-VQR (Addgene #159982), and pCMV-PE2-SpG (Addgene #159978) were obtained from Addgene, Watertown, MA, USA (https://www.addgene.org/). These plasmids encode a fusion protein consisting of an engineered SpCas9 H840A nickase and an M-MLV reverse transcriptase, enabling precise genome editing without generating double-strand DNA breaks.

The pU6-tevopreq1-GG-acceptor plasmid (Addgene #174038), which allows U6 promoter-driven expression of enhanced prime editing guide RNAs (epegRNAs), was also obtained from Addgene. In our laboratory, this plasmid was further modified to include an additional U6 promoter and a cloning site for expression of a nicking single-guide RNA (nsgRNA) required for the PE3 strategy. This modified construct was designated pU6-epegRNA-nsgRNA.

Design of epegRNAs and nsgRNAs was performed according to the guidelines described by Anzalone et al. [12], with optimization of primer binding site (PBS) and reverse transcription template (RTT) lengths to maximize editing efficiency. Particular attention was given to avoiding premature transcriptional termination motifs within the epegRNA scaffold. All oligonucleotides used for cloning were synthesized by Integrated DNA Technologies (IDT, Coralville, IA, USA). Cloning was performed using standard restriction enzyme digestion and ligation or Golden Gate assembly, followed by sequence verification.

### 2.3. Cell Culture Conditions

C2C12 myoblasts were cultured in Dulbecco’s Modified Eagle Medium (DMEM; Wisent Inc., Saint-Jean-Baptiste, QC, Canada) supplemented with 10% fetal bovine serum (FBS) and 1% penicillin–streptomycin (both from Wisent Inc.). Cells were maintained at 37 °C in a humidified incubator with 5% CO_2_. Culture medium was replaced every 2–3 days, and cells were passaged at approximately 70–80% confluence using standard trypsinization procedures. All experiments were performed under sterile conditions.

### 2.4. Plasmid Electroporation in Mouse C2C12 Myoblasts

Plasmid delivery into C2C12 myoblasts was performed using the Neon™ Transfection System (Thermo Fisher Scientific, Waltham, MA, USA). Briefly, 100,000 cells were resuspended in Neon resuspension buffer and electroporated with a total of 2 μg of plasmid DNA per reaction, consisting of 1 μg of a prime editor plasmid (PE2, PE6a, PE-VQR, or PE-SpG) and 1 μg of the corresponding epegRNA-nsgRNA plasmid (for PE3 experiments).

Electroporation was carried out using 10 μL Neon tips with the following parameters: 1300 V, 20 ms pulse width, and 2 pulses. Immediately after electroporation, the cells were transferred into one well of a 24-well plate containing 500 μL of pre-warmed culture medium. After 24 h, the medium was replaced with 1 mL of fresh DMEM to remove cell debris and residual electroporation buffer. Cells were incubated for an additional 48 h to allow sufficient time for prime editing to occur.

As a negative control, cells were electroporated with a plasmid expressing enhanced green fluorescent protein (eGFP) alone. Transfection efficiency was estimated by fluorescence microscopy and consistently exceeded 80%.

### 2.5. Positive and Negative Control Constructs

An epegRNA targeting the mouse *Atp2a2* locus was used as a positive control for prime editing activity. This target was selected based on prior internal optimization experiments demonstrating reproducibly high editing efficiency under similar electroporation and plasmid delivery conditions. Editing efficiency at the Atp2a2 locus was evaluated using Sanger sequencing followed by EditR online program (https://moriaritylab.shinyapps.io/editr_v10/, accessed on 1 March 2024 to February, 1 September 2025), consistent with the quantification method used for *Dmd* loci.

For negative control experiments and assessment of electroporation efficiency, an engineered eGFP-expressing plasmid was used. Transfection efficiency was determined by fluorescence microscopy based on the proportion of GFP-positive cells, which routinely exceeded ~80% under optimized electroporation conditions. In this control condition, no *Dmd*-targeting guide RNA was included. Background signal at the *Dmd* locus was assessed by Sanger sequencing to confirm the absence of detectable editing events. All control and experimental plasmids were transfected at equal total DNA amounts (2 μg per reaction) to ensure consistency across conditions.

### 2.6. Genomic DNA Extraction and PCR Amplification

Genomic DNA was extracted directly from cultured C2C12 cells using the DirectPCR™ Lysis Reagent (Viagen Biotech Inc., Los Angeles, CA, USA). Cells were washed once with 500 μL phosphate-buffered saline (PBS), followed by addition of 100 μL lysis reagent supplemented with 1 μL proteinase K (20 mg/mL). Samples were incubated at 56 °C for 2 h to ensure complete lysis and protein digestion, followed by enzyme inactivation at 85 °C for 45 min.

**Figure 3:**
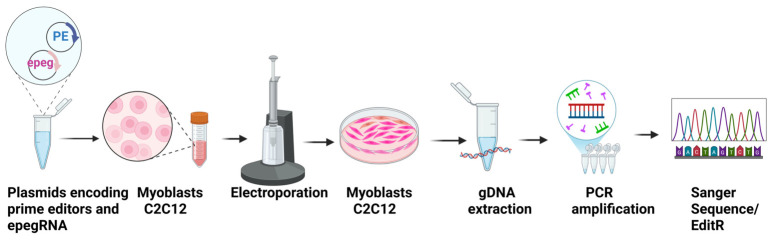
**Methodology.** Plasmids encoding PE and epegRNA were first constructed by cloning. The plasmids were then transfected into C2C12 cells by Neon electroporation. 72 h after transfecting the cells, the gDNA of these cells was extracted. Part of the targeted gene was amplified by PCR, then sequenced by Sanger sequencing. The percentage of editing obtained was determined using EditR software. Image created with https://BioRender.com.

Lysates were centrifuged at 13,000 rpm for 5 min, and 1–2 μL of the supernatant was used as template DNA for PCR amplification of the target genomic regions. PCR was performed using Phusion™ High-Fidelity DNA Polymerase (Thermo Fisher Scientific) to minimize amplification errors. Cycling conditions consisted of an initial denaturation at 98 °C for 30 s, followed by 35 cycles of 98 °C for 10 s, 62 °C for 20 s, and 72 °C for 30 s, with a final extension step at 72 °C for 30 s. Primer sequences were designed to flank the edited region and generate amplicons suitable for Sanger sequencing (Figure 3).

### 2.7. Sanger Sequencing and Editing Analysis

PCR products were submitted for Sanger sequencing to the CHU de Québec Research Center sequencing platform, Université Laval, Québec, Canada (https://sequences.ulaval.ca/murin/servseq.pageaccueil) between 1 March 2024 and 28 February 2025. Sequencing reactions were performed using BigDye™ Terminator v3.1 chemistry (Thermo Fisher Scientific) with internal primers to ensure high-quality base calling across the edited site (Figure 3).

Although amplicon deep sequencing provides higher sensitivity and quantitative resolution, Sanger sequencing combined with EditR analysis is a validated and widely used approach for estimating editing frequencies in initial optimization studies [31,32]. Sequencing chromatograms were analyzed to quantify prime editing efficiencies using the EditR online tool (https://moriaritylab.shinyapps.io/editr_v10/) accessed from 1 March 2024 to 28 February 2025, which enables robust estimation of nucleotide substitution frequencies from mixed Sanger traces. Editing efficiency was calculated as the percentage of modified alleles relative to the total signal at the target nucleotide position.

### 2.8. Statistical Analysis

All quantitative data were analyzed using GraphPad Prism version 10.4.1 (GraphPad Software Inc., La Jolla, CA, USA). Comparisons of editing efficiencies among multiple experimental groups were performed using the nonparametric Kruskal–Wallis test. Data are presented as mean ± standard error of the mean (SEM). A *p*-value < 0.05 was considered statistically significant, corresponding to a 95% confidence level. A n values represent independent biological replicates, defined as separate C2C12 cell cultures of the same cell line, each subjected to the full experimental workflow independently, including electroporation, culture, DNA extraction, and sequencing. Each experiment was performed under identical conditions to ensure reproducibility.

## 3. Results

### 3.1. Installation of the 4cv Nonsense Mutation in Exon 53 of the Dmd Gene in C2C12 Myoblasts Using Prime Editing

To validate the feasibility of using prime editing to precisely modify single nucleotides within the *Dmd* gene, we first sought to introduce the 4cv nonsense mutation into exon 53 of the *Dmd* gene in vitro. This approach served as a proof-of-concept to establish whether prime editing could efficiently generate a disease-relevant point mutation in myogenic cells prior to attempting mutation correction.

**Figure 4:**
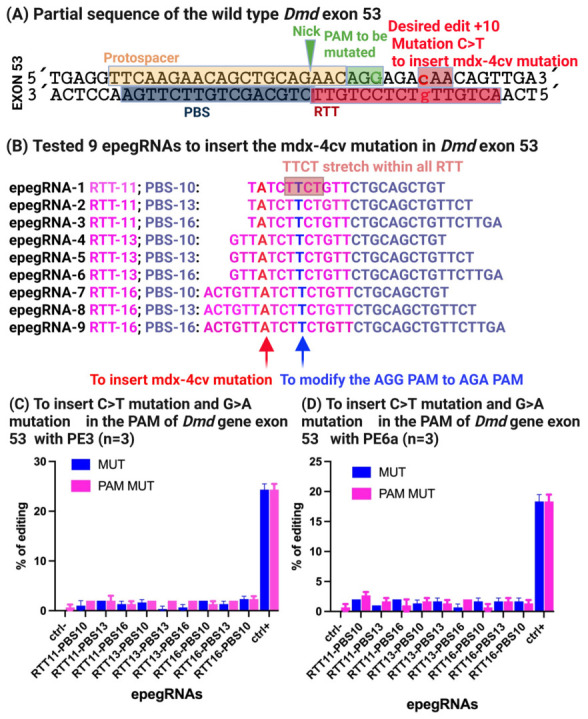
**Generation of the mdx-4cv mutation in *Dmd* exon 53 by prime editing and identification of epegRNA design constraints.** (**A**) Schematic representation of the prime editing strategy used to introduce the mdx-4cv nonsense mutation (CAA → TAA) in exon 53 of the *Dmd* gene using SpCas9 nickase (H840A) recognizing a nearby NGG (5′-AGG-3′) PAM. The epegRNA design included a silent PAM-disrupting mutation to prevent re-editing. (**B**) Design of nine epegRNAs differing in reverse transcription template (RTT) and primer binding site (PBS) lengths. All constructs were found to contain an unintended TTCT sequence within the RTT. (**C**,**D**) Quantification of editing efficiency in wild-type C2C12 myoblasts using PE3 and PE6a three days post-electroporation, assessed by Sanger sequencing and EditR analysis. Limited editing (~2%) was detected at the target nucleotide, with minimal PAM modification, indicating suboptimal prime editing efficiency.

A target site within exon 53 harboring a proximal NGG PAM (5′-AGG-3′) was selected, enabling the use of the SpCas9 nickase (H840A) fused to an M-MLV reverse transcriptase. The prime editing strategy was designed to convert the wild-type CAA codon encoding glutamine into a TAA stop codon, thereby recreating the 4cv mutation observed in the mdx-4cv mouse model (Figure 4A).

To optimize editing efficiency, nine distinct engineered prime editing guide RNAs (epegRNAs) incorporating the tevopreQ1 motif at the 3′ end were designed to protect the PBS and RTT sequences from exonuclease-mediated degradation [14] were designed, differing in the lengths of their primer binding sites (PBS) and reverse transcription templates (RTT) (Figure 4B). In addition to introducing the nonsense mutation, a silent PAM-disrupting mutation was incorporated to prevent repeated Cas9 recognition following successful editing and to potentially enhance product stability.

Prime editing was performed by electroporating C2C12 mouse myoblasts, which carry a wild-type *Dmd* locus, with either pCMV-PE2 or pCMV-PE6a together with the corresponding pU6-pegRNA-GG-acceptor vector expressing individual epegRNAs. Negative control cells were electroporated with a plasmid encoding eGFP alone to monitor transfection efficiency and background editing.

Genomic DNA was extracted 72 h post-electroporation, and editing outcomes were assessed by Sanger sequencing, followed by quantitative analysis using the EditR software [32]. Across all nine tested epegRNAs, only low-level editing efficiencies were observed, with a maximum of approximately 2% conversion of the target guanine to thymine and ~1% incorporation of the silent PAM mutation (Figure 4C).

Despite the favorable proximity of the nicking site to the intended edit (+10 nucleotides) and the inclusion of a PAM-disrupting mutation, editing efficiency remained unexpectedly low. To understand this limitation, we examined the epegRNA sequences in detail and identified the presence of a 5′-TTCT-3′ stretch within the RTT region of all nine constructs (Figure 4B, Supplementary Table S1). Previous studies have shown that such sequences can cause premature transcriptional termination of U6-driven guide RNAs, thereby severely reducing functional pegRNA expression and prime editing efficiency [33,34]. In the subsequent experiments, we did 9 epegRNA with optimatizations of the RTT sequence to remove this 5′-TTCT-3′ stretch.

### 3.2. Optimization of epegRNAs by Removal of the TTCT Stretch Enables Efficient Correction of the 4cv Mutation In Vitro

To overcome the transcriptional limitation identified in the initial designs, we next optimized epegRNAs to remove the 5′-TTCT-3′ stretch while maintaining precise correction ~20% of the 4cv nonsense mutation. The optimized strategy aimed to convert the TAA stop codon back to the wild-type CAA glutamine codon in exon 53 of the *Dmd* gene.

**Figure 5:**
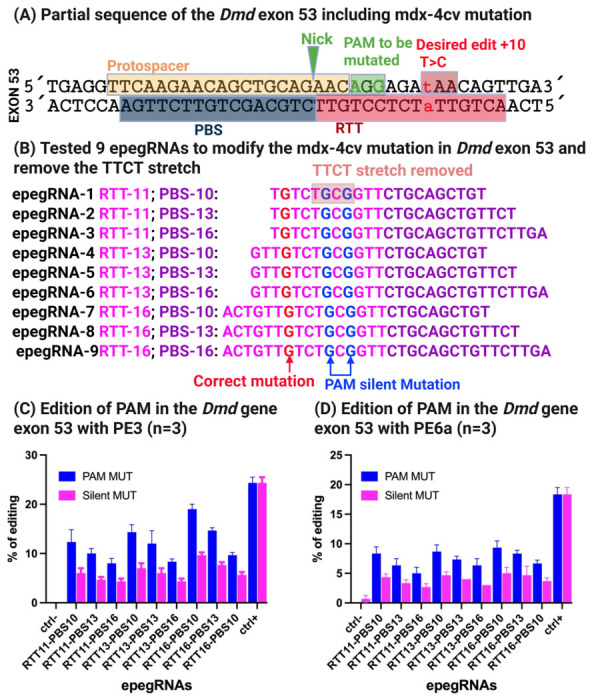
**Optimization of epegRNA design enables efficient correction of the mdx-4cv mutation in *Dmd* exon 53.** (**A**) Strategy for correcting the mdx-4cv mutation (TAA → CAA) using redesigned epegRNAs targeting the same NGG PAM site. Two synonymous PAM mutations (AGG → CGC) encoding arginine were introduced to remove the TTCT stretch and enable detection of editing in wild-type cells. (**B**) Updated epegRNA architectures showing optimized RTT and PBS configurations lacking the TTCT motif. (**C**,**D**) Editing efficiencies obtained in wild-type C2C12 myoblasts using PE3 and PE6a. The epegRNA-16-10 construct achieved the highest correction efficiency (20% with PE3 and 6% with PE6a). An Atp2a2-targeting epegRNA served as a positive control, while no background editing was observed in negative controls.

Nine newly designed epegRNAs were generated, all targeting the same NGG PAM (5′-AGG-3′). To eliminate the 5′-TTCT-3′ motif and simultaneously improve experimental readout, we introduced two silent mutations within the PAM, converting AGG to CGC, both of which encode arginine (Figure 5A,B). Importantly, this modification served two purposes:

(a) Removal of the 5′-TTCT-3′ sequence to restore proper epegRNA transcription.

(b) Introduction of a traceable silent mutation, allowing editing efficiency to be quantified even in wild-type C2C12 cells lacking the pathogenic stop codon.

Prime editing experiments were conducted in wild-type C2C12 myoblasts using PE3 and PE6a prime editing techniques, and editing efficiency was evaluated by quantifying incorporation of the silent PAM mutation. An epegRNA targeting the Atp2a2 gene was included as a positive control to confirm overall prime editing activity.

Among the nine optimized epegRNAs, epegRNA-16-10, containing a 16-nucleotide RTT and a 10-nucleotide PBS, demonstrated the highest editing efficiency. Following a single electroporation, this construct achieved 20% editing with PE3 and 6% editing with PE6a (Figure 5C). In comparison, the positive control Atp2a2 epegRNA resulted in 23% editing with PE3 and 19% with PE6a, while no detectable background editing was observed in negative control samples.

These results demonstrate that removal of the 5′-TTCT-3′ motif markedly improves prime editing efficiency, validating the optimized epegRNAs design and confirming the feasibility of correcting the 4cv mutation in vitro.

### 3.3. In Vitro Prime Editing of the 5cv Mutation Using an NGAG PAM

We next extended our prime editing strategy to the 5cv mutation in the *Dmd* gene. This mutation involves a single-nucleotide substitution in exon 10, where the wild-type GGA glycine codon is converted to GGT, creating an aberrant splice donor site that leads to dystrophin dysfunction.

**Figure 6:**
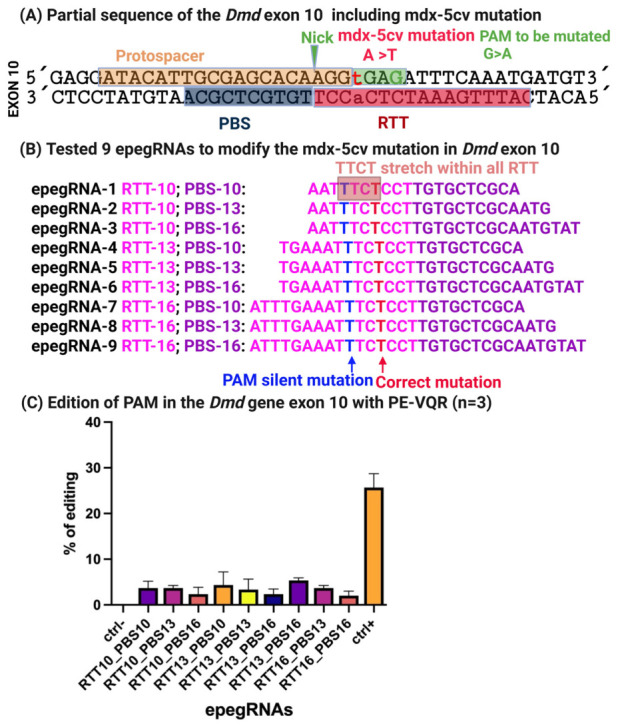
**Prime editing-mediated targeting of the mdx-5cv mutation using an NGAG PAM and SpCas9-VQR.** (**A**) Illustration of the mdx-5cv mutation in exon 10, where a synonymous GGA → GGT substitution creates a cryptic splice donor site. (**B**) Design of nine epegRNAs targeting the mutation using SpCas9-VQR recognizing a 5′-NGAG-3′ PAM. RTT and PBS lengths were systematically varied, and a silent PAM mutation was included to allow detection of editing in wild-type cells. All constructs contained a TTCT stretch. (**C**) Editing efficiencies measured in C2C12 myoblasts following electroporation. Targeted editing ranged from 2–6%, whereas the Atp2a2 positive control reached 29% efficiency. Transfection efficiency exceeded 80%, as confirmed by eGFP expression.

To target this locus, we designed nine epegRNAs with variable RTT and PBS lengths (Figure 6A,B). Because no suitable NGG PAM was present near the mutation, we selected the SpCas9-VQR nickase variant, which recognizes a 5′-NGAG-3′ PAM, enabling precise targeting of the 5cv locus [35].

Since C2C12 cells contain the wild-type GGA codon and do not harbor the disease mutation, a silent PAM mutation was incorporated into the design to allow detection of prime editing events independently of mutation correction. C2C12 myoblasts were electroporated with PE-VQR and individual epegRNAs, while negative controls received an eGFP reporter plasmid. Transfection efficiency was consistently high, exceeding 80%.

Editing outcomes were assessed by Sanger sequencing. Across the nine epegRNAs tested, editing efficiencies ranged from 2% to 6% at the target locus. In contrast, the Atp2a2 positive control achieved 29% editing, confirming robust prime editing activity under the same experimental conditions (Figure 6C). Background editing in negative controls remained negligible.

Sequence analysis of the epegRNAs revealed that, similar to the initial 4cv designs, all nine constructs contained a 5′-TTCT-3′ motif within the RTT (Figure 6B), likely accounting for the limited editing efficiency. This finding reinforced the importance of avoiding transcription-terminating sequences in epegRNA design and motivated further optimization [33]. In the subsequent experiments, we did 9 epegRNA optimatizations of the RTT and PBS to remove this 5′-TTCT-3′ stretch.

### 3.4. Removal of the TTCT Stretch Enhances Prime Editing Efficiency for Modification of the 5cv Mutation In Vitro

**Figure 7:**
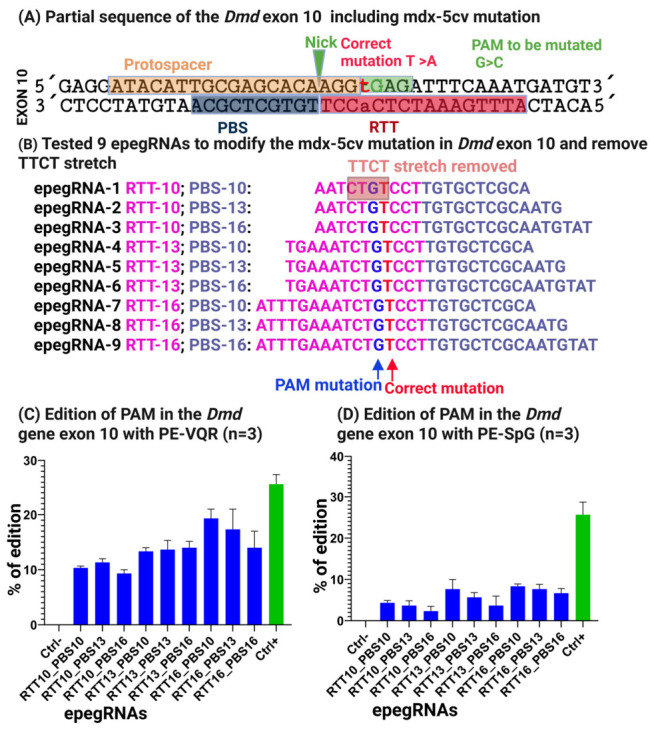
**Removal of the TTCT stretch markedly improves prime editing efficiency for correction of the mdx-5cv mutation.** (**A**) Schematic of the optimization strategy in which a PAM-disrupting nucleotide substitution was introduced to eliminate the TTCT motif, resulting in a conservative amino-acid change (glutamic acid to glutamine) while preserving protein function and preventing Cas9 re-binding to the edited allele. (**B**) Final optimized epegRNA designs targeting NGAG protospacer adjacent motifs (PAMs) recognized by SpCas9-VQR or NGN PAMs recognized by SpCas9-spG. (**C**,**D**) Quantitative analysis of prime-editing outcomes in C2C12 myoblasts. Optimized epegRNAs achieved editing efficiencies of up to 21% using PE-VQR and 9% using PE-spG. Editing at the Atp2a2 locus served as a positive control and reached 29% efficiency, confirming robust prime-editing activity.

To improve prime editing efficiency at the 5cv mutation site, epegRNAs were redesigned to disrupt a 5′-TTCT-3′ sequence located within the reverse transcription template (RTT). This was achieved by introducing a nucleotide substitution with a conservative amino acid change, converting glutamic acid to glutamine (E → Q). This substitution preserves the physicochemical properties of the protein while effectively eliminating the 5′-TTCT-3′ sequence from the RTT (Figure 7A,B).

The optimized epegRNAs were designed to target either 5′-NGAG-3′ PAMs using the VQR variant [35] or 5′-NGN-3′ PAMs using the SpG variant [36]. All constructs were cloned into the pU6-epegRNA-GG-acceptor plasmid and delivered into C2C12 cells by electroporation together with plasmids coding either for pCMV-PE2-VQR or pCMV-PE2-SpG [36]. Negative controls expressing eGFP confirmed transfection efficiencies of at least 80%.

Following optimization, prime editing efficiency increased substantially. Editing at the 5cv target site reached 21% with PE-VQR and 9% with PE-spG (Figure 7C). In parallel, the Atp2a2 positive control again achieved 29% editing, confirming consistent prime editing performance across experiments.

Collectively, these results demonstrate that strategic elimination of transcription-terminating sequence motifs within epegRNAs significantly improves prime editing efficiency and provides practical design guidance for optimizing editing outcomes. The optimized reagents identified in this study establish a foundation for future validation in disease-relevant systems, including primary cells derived from mdx-4cv and mdx-5cv mouse models, as well as in vivo studies aimed at evaluating the therapeutic potential of prime editing for DMD-associated mutations.

## 4. Discussion

In this study, we establish efficient prime editing-mediated modification of two point mutations and define key guide RNA design parameters that govern editing efficiency in myogenic cells. By systematically optimizing engineered prime editing guide RNAs (epegRNAs) for the mdx-4cv and mdx-5cv loci, we achieved editing efficiencies of up to 20–21% levels at the genomic DNA level in C2C12 myoblasts, demonstrating robust editing activity under in vitro conditions. Wild-type C2C12 myoblasts were employed as a design-optimization model rather than a disease model, providing a reproducible platform for evaluating prime editing efficiency and guide RNA performance. Future studies using primary cells derived from mdx-4cv or mdx-5cv mice will be necessary to validate the therapeutic applicability of these optimized prime editing strategies in disease-relevant cellular contexts.

Our data demonstrate that prime editing outcomes at the *Dmd* locus are strongly influenced by the combined length and composition of the reverse transcription template (RTT) and primer binding site (PBS). In the case of the mdx-4cv mutation, nine initial epegRNA designs spanning multiple RTT and PBS lengths resulted in uniformly low editing efficiencies (≤2%), despite optimal PAM proximity and high transfection efficiency (Figure 4C). These results underscore that favorable genomic targeting alone is insufficient to ensure productive prime editing.

Following redesign to eliminate the 5′-TTCT-3′ motif, editing efficiencies increased markedly and revealed clear differences among RTT–PBS configurations. Notably, the epegRNA-16-10 construct, containing a 16-nucleotide RTT and a 10-nucleotide PBS, consistently outperformed other designs, achieving ~20% editing with PE3 and ~6% with PE6a (Figure 5C). This observation is consistent with prior reports indicating that intermediate-length PBS and RTT combinations often provide optimal primer annealing and reverse transcription efficiency without compromising flap resolution or DNA repair outcomes [12,35].

Importantly, the lower editing efficiency observed with PE6a compared to PE3 at the same locus suggests that nicking of the non-edited strand remains a significant contributor to productive editing at the mdx-4cv site. This is in agreement with previous studies demonstrating that PE3 frequently outperforms PE2- or PE6-based strategies at loci where mismatch repair bias favors reversion of the edited strand.

A central finding of this work is the identification of a short 5′-TTCT-3′ motif within the RTT that severely limits prime editing efficiency when epegRNAs are expressed under a U6 promoter. While RNA polymerase III termination has classically been associated with poly-T tracts (≥4 thymidines), accumulating evidence indicates that shorter thymidine-rich motifs can also impair transcriptional processivity or guide RNA stability [37,38]. Our data extend this concept by demonstrating that the 5′-TTCT-3′ motif, although shorter than canonical terminators, is sufficient to reduce functional epegRNA output in a biologically relevant context.

Crucially, elimination of the 5′-TTCT-3′ motif markedly improved prime editing efficiencies, increasing them from ≤2% to ~20% at the mdx-4cv locus and from ≤6% to ~21% at the mdx-5cv locus. This enhancement was reproducible across multiple prime editor architectures, indicating that the improvement reflects increased epegRNA performance rather than editor-specific effects. Notably, additional adjustments to RTT/PBS configurations and PAM codons were also implemented, suggesting that the observed gains likely arise from the combined influence of these design optimizations rather than the TTCT motif removal alone.

Modification of the mdx-5cv mutation posed additional challenges due to the absence of a nearby NGG PAM. By employing the SpCas9-VQR variant, which recognizes NGAG PAMs, we were able to access this clinically relevant locus and achieve initial editing efficiencies of 2–6% across nine epegRNA designs (Figure 6C). The relatively modest editing levels observed at this stage mirrored those seen in the initial mdx-4cv experiments and again correlated with the presence of the 5′-TTCT-3′ motif in all RTT designs.

Following redesign of the RTT to eliminate the 5′-TTCT-3′ motif through a conservative glutamic acid-to-glutamine substitution, editing efficiency increased substantially. Using PE-VQR, optimized epegRNAs achieved ~21% editing, whereas PE-spG resulted in ~9% editing at the same locus (Figure 7C). These results demonstrate that PAM expansion alone is not sufficient to ensure efficient prime editing and that epegRNA sequence integrity remains a dominant determinant of editing success.

The difference in performance between PE-VQR and PE-SpG further highlights locus-specific constraints in prime editing, likely reflecting differences in PAM binding affinity, nick positioning, and downstream repair outcomes.

## 5. Implications for Therapeutic Prime Editing of DMD

From a therapeutic perspective, the editing efficiencies achieved in this study are notable. Previous work has demonstrated that partial restoration of dystrophin expression can confer substantial functional benefit in Duchenne muscular dystrophy (DMD), with dystrophin levels in the range of approximately 10–20% often sufficient to produce measurable improvements in muscle pathology and function in both animal models and patients [39,40,41]. Genome editing studies have further shown that incomplete correction leading to partial dystrophin recovery can significantly ameliorate disease phenotypes [42,43]. Although the present study is limited to in vitro modification in myoblasts, the optimized epegRNA designs and editor configurations established here provide a strong foundation for subsequent in vitro and in vivo correction studies.

More broadly, our findings highlight that epegRNA design must consider not only target sequence selection and RTT–PBS optimization, but also transcriptional constraints imposed by RNA polymerase III promoters. Pol III– driven guide RNAs are known to be sensitive to short thymidine-rich termination motifs, which can lead to premature transcriptional termination [44]. The identification of a 5′-TTCT-3′ motif as a functional design pitfall in this study therefore has practical implications and is likely relevant to a wide range of prime editing applications beyond DMD. While we achieved prime editing efficiencies of up to ~20–21% at the DNA level in proliferating C2C12 myoblasts, these modifications were performed in wild-type rather than mutant cells; therefore, the observed efficiencies do not directly translate into proportional dystrophin protein restoration. Dystrophin expression and functional recovery depend on additional factors, including myoblast differentiation into multinucleated myotubes, RNA processing, and protein stability. Future studies will focus on correcting this mutation in primary cell lines and/or engineered mutant cell lines, as well as evaluating protein-level outcomes in differentiated myotubes and in vivo models, to fully assess the therapeutic potential of these optimized prime editing strategies.

## 6. Conclusions

In summary, we demonstrate efficient prime editing-mediated edition of the mdx-4cv and mdx-5cv mutations and identify a critical, previously underappreciated guide RNA design constraint that governs editing efficiency. By systematically optimizing RTT–PBS configurations and eliminating a cryptic transcription termination motif, we achieved robust editing efficiencies of up to 20–21% across multiple prime editor variants. These findings provide important design principles for therapeutic prime editing and support the feasibility of precise genome correction for Duchenne muscular dystrophy.

## Data Availability

The original contributions presented in this study are included in the article/Supplementary Materials. Further inquiries can be directed to the corresponding authors.

## Supplementary Materials

The following supporting information can be downloaded at: https://www.mdpi.com/article/10.3390/cells15090740/s1. Supporting Table S1. epegRNA sequences.
