# Supplementary material for: Successful In Vitro Modification of the Dmd Gene Using Prime Editing
Source: Cells. 2026 Apr 22;15(9):740. doi: 10.3390/cells15090740 (PMC13162614; doi:10.3390/cells15090740)
Supplement: Supplementary file 1 [file cells-15-00740-s001.zip › cells-4177768-supplementary.pdf]

Supporting Table S1. epegRNA sequences				
**epegRNA sequences to create 4CV mutation**				
**Names**	**Spacer sequence**	**PBS sequences**	**RTT sequences**	**nsgRNA for PE3**
epegRNA1	gTTCAAGAACAGCTGCAGAAC	CTGCAGCTGT	TATCTTCTGTT	GACCTGTCCTATGACCT GTT
epegRNA2	gTTCAAGAACAGCTGCAGAAC	CTGCAGCTGTTCT	TATCTTCTGTT	GACCTGTCCTATGACCT GTT
epegRNA3	gTTCAAGAACAGCTGCAGAAC	CTGCAGCTGTTCTT GA	TATCTTCTGTT	GACCTGTCCTATGACCT GTT
epegRNA4	gTTCAAGAACAGCTGCAGAAC	CTGCAGCTGT	GTTATCTTCTGTT	GACCTGTCCTATGACCT GTT
epegRNA5	gTTCAAGAACAGCTGCAGAAC	CTGCAGCTGTTCT	GTTATCTTCTGTT	GACCTGTCCTATGACCT GTT
epegRNA6	gTTCAAGAACAGCTGCAGAAC	CTGCAGCTGTTCTT GA	GTTATCTTCTGTT	GACCTGTCCTATGACCT GTT
epegRNA7	gTTCAAGAACAGCTGCAGAAC	CTGCAGCTGT	ACTGTTATCTTCTG TT	GACCTGTCCTATGACCT GTT
epegRNA8	gTTCAAGAACAGCTGCAGAAC	CTGCAGCTGTTCT	ACTGTTATCTTCTG TT	GACCTGTCCTATGACCT GTT
epegRNA9	gTTCAAGAACAGCTGCAGAAC	CTGCAGCTGTTCTT GA	ACTGTTATCTTCTG TT	GACCTGTCCTATGACCT GTT
**Modified epegRNA sequences for the correction of 4CV mutation**				
epegRNA 1	gTTCAAGAACAGCTGCAG AAC	CTGCAGCTGT	TGTCTGCGGTT	GACCTGTCCTATGACCT GTT
epegRNA 2	gTTCAAGAACAGCTGCAG AAC	CTGCAGCTGTTCT	TGTCTGCGGTT	GACCTGTCCTATGACCT GTT
epegRNA 3	gTTCAAGAACAGCTGCAG AAC	CTGCAGCTGTTCTTGA	TGTCTGCGGTT	GACCTGTCCTATGACCT GTT
epegRNA 4	gTTCAAGAACAGCTGCAG AAC	CTGCAGCTGT	GTTGTCTGCGGTT	GACCTGTCCTATGACCT GTT
epegRNA 5	gTTCAAGAACAGCTGCAG AAC	CTGCAGCTGTTCT	GTTGTCTGCGGTT	GACCTGTCCTATGACCT GTT
epegRNA 6	gTTCAAGAACAGCTGCAG AAC	CTGCAGCTGTTCTTGA	GTTGTCTGCGGTT	GACCTGTCCTATGACCT GTT
epegRNA 7	gTTCAAGAACAGCTGCAG AAC	CTGCAGCTGT	ACTGTTGTCTGCGGTT	GACCTGTCCTATGACCT GTT
epegRNA 8	gTTCAAGAACAGCTGCAG AAC	CTGCAGCTGTTCT	ACTGTTGTCTGCGGTT	GACCTGTCCTATGACCT GTT
epegRNA 9	gTTCAAGAACAGCTGCAG AAC	CTGCAGCTGTTCTTGA	ACTGTTGTCTGCGGTT	GACCTGTCCTATGACCT GTT
**epegRNA sequences for the creation of 5CV mutation (NGG PAM)**				
epegRNA 1	GAGGATACATTGCGAGCAC A	GCTCGCAATG	CTCACCTTGT	GTACTTCTTCTAAAGCA GTT
epegRNA 2	GAGGATACATTGCGAGCAC A	GCTCGCAATGTAT	CTCACCTTGT	GTACTTCTTCTAAAGCA GTT
epegRNA 3	GAGGATACATTGCGAGCAC A	GCTCGCAATGTATCCT	CTCACCTTGT	GTACTTCTTCTAAAGCA GTT

Table S1. epegRNA sequences				
**Name**	**Spacer sequence**	**PBS sequences**	**RTT sequences**	**nsgRNA for PE3**
epegRNA 4	GAGGATACATTGCGAGCACA	GCTCGCAATG	AATCTCACCTTGT	GTACTTCTTCTAAAGCAGTT
epegRNA 5	GAGGATACATTGCGAGCACA	GCTCGCAATGTAT	AATCTCACCTTGT	GTACTTCTTCTAAAGCAGTT
epegRNA 6	GAGGATACATTGCGAGCACA	GCTCGCAATGTATCCT	AATCTCACCTTGT	GTACTTCTTCTAAAGCAGTT
epegRNA 7	GAGGATACATTGCGAGCACA	GCTCGCAATG	TTGAAATCTCACCTTGT	GTACTTCTTCTAAAGCAGTT
epegRNA 8	GAGGATACATTGCGAGCACA	GCTCGCAATGTAT	TTGAAATCTCACCTTGT	GTACTTCTTCTAAAGCAGTT
epegRNA 9	GAGGATACATTGCGAGCACA	GCTCGCAATGTATCCT	TTGAAATCTCACCTTGT	GTACTTCTTCTAAAGCAGTT
**epegRNA sequences for the correction of 5CV mutation (NGAG PAM)**				
epegRNA 1	GATACATTGCGAGCACAAGG	TGTGCTCGCA	AATTTCTCCT	CAGTGTTTTCTTTTACCTCA
epegRNA 2	GATACATTGCGAGCACAAGG	TGTGCTCGCAATG	AATTTCTCCT	CAGTGTTTTCTTTTACCTCA
epegRNA 3	GATACATTGCGAGCACAAGG	TGTGCTCGCAATGTAT	AATTTCTCCT	CAGTGTTTTCTTTTACCTCA
epegRNA 4	GATACATTGCGAGCACAAGG	TGTGCTCGCA	TGAAATTTCTCCT	CAGTGTTTTCTTTTACCTCA
epegRNA 5	GATACATTGCGAGCACAAGG	TGTGCTCGCAATG	TGAAATTTCTCCT	CAGTGTTTTCTTTTACCTCA
epegRNA 6	GATACATTGCGAGCACAAGG	TGTGCTCGCAATGTAT	TGAAATTTCTCCT	CAGTGTTTTCTTTTACCTCA
epegRNA 7	GATACATTGCGAGCACAAGG	TGTGCTCGCA	TAAACTTTAAAGAGGA	CAGTGTTTTCTTTTACCTCA
epegRNA 8	GATACATTGCGAGCACAAGG	TGTGCTCGCAATG	TAAACTTTAAAGAGGA	CAGTGTTTTCTTTTACCTCA
epegRNA 9	GATACATTGCGAGCACAAGG	TGTGCTCGCAATGTAT	TAAACTTTAAAGAGGA	CAGTGTTTTCTTTTACCTCA
**Modified epegRNA sequences for the correction of 5CV mutation (NGAG PAM)**				
epegRNA 1	GATACATTGCGAGCACAAGG	TGTGCTCGCA	AATCTGTCCT	CAGTGTTTTCTTTTACCTCA
epegRNA 2	GATACATTGCGAGCACAAGG	TGTGCTCGCAATG	AATCTGTCCT	CAGTGTTTTCTTTTACCTCA
epegRNA 3	GATACATTGCGAGCACAAGG	TGTGCTCGCAATGTAT	AATCTGTCCT	CAGTGTTTTCTTTTACCTCA
epegRNA 4	GATACATTGCGAGCACAAGG	TGTGCTCGCA	TGAAATCTGTCCT	CAGTGTTTTCTTTTACCTCA
epegRNA 5	GATACATTGCGAGCACAAGG	TGTGCTCGCAATG	TGAAATCTGTCCT	CAGTGTTTTCTTTTACCTCA
epegRNA 6	GATACATTGCGAGCACAAGG	TGTGCTCGCAATGTAT	TGAAATCTGTCCT	CAGTGTTTTCTTTTACCTCA
epegRNA 7	GATACATTGCGAGCACAAGG	TGTGCTCGCA	ATTTGAAATCTGTCCT	CAGTGTTTTCTTTTACCTCA
epegRNA 8	GATACATTGCGAGCACAAGG	TGTGCTCGCAATG	ATTTGAAATCTGTCCT	CAGTGTTTTCTTTTACCTCA

**Table S1. epegRNA sequences**				
**Name**	**Spacer sequence**	**PBS sequences**	**RTT sequences**	**nsgRNA for PE3**
epegRNA 9	GATACATTGCGAGCACAAGG	TGTGCTCGCAATGTAT	ATTTGAAATCTGTCCT	CAGTGTTTTCTTTTACCTCA
